# An unusual case of *Cardiobacterium valvarum* causing aortic endograft infection and osteomyelitis

**DOI:** 10.1186/s12941-021-00419-w

**Published:** 2021-02-27

**Authors:** Eric G. Hauser, Imran Nizamuddin, Brett B. Yarusi, Karen M. Krueger

**Affiliations:** 1 Department of Medicine, Feinberg School of Medicine, Northwestern University, Chicago, IL USA; 2 Division of Infectious Diseases, Feinberg School of Medicine, Northwestern University, Chicago, IL USA

**Keywords:** *Cardiobacterium valvarum*, Infective endocarditis, Aortic endograft infection, HACEK organisms

## Abstract

**Background:**

HACEK (*Haemophilus* spp., *Aggregatibacter* spp., *Cardiobacterium* spp., *Eikenella corrodens*, and *Kingella* spp.) group organisms are responsible for 0.8% to 6% of all infective endocarditis cases, with *Cardiobacterium *spp. being the third most commonly implicated HACEK microorganism. Within this genus is *Cardiobacterium valvarum* (*C. valvarum*), a novel organism described in 2004. To date, only 15 cases of *C. valvarum* infection have been reported in the English-language literature, and have primarily been cases of infective endocarditis in patients with valvular disease. *C. valvarum* has not been reported to cause infections spreading to the surrounding bone.

**Case presentation:**

We present a case of a 57-year-old man with a history of aortic dissection followed by aortic endograft replacement who presented with back pain. He was found to have radiographic evidence of an infected aortic endograft, along with vertebral osteomyelitis, discitis, and epidural phlegmon. Blood cultures identified *C. valvarum* as the causative organism. The patient was treated with ceftriaxone and surgical intervention was deferred due to the patient’s complex anatomy. His course was complicated by septic cerebral emboli resulting in cerebrovascular accident.

**Conclusions:**

This case report highlights *C. valvarum*, a rare and emerging HACEK group microorganism that warrants consideration in high-risk patients with evidence of subacute infection and disseminated disease. While *C. valvarum* classically presents as infective endocarditis, extra-cardiac manifestations have also been described. As demonstrated in this case, endograft involvement and osteomyelitis may occur in rare circumstances.

## Background

*Cardiobacterium* spp. are fastidious, pleomorphic Gram-negative bacilli that belong to the HACEK (*Haemophilus* spp., *Aggregatibacter* spp., *Cardiobacterium* spp., *Eikenella corrodens*, and *Kingella* spp.) group of microorganisms, a group of bacteria that share similar microbiological and clinical characteristics. Species within this group have been reported to be responsible for 0.8–6.0% of all infective endocarditis cases [1]. Although an uncommon cause of endocarditis overall, *Cardiobacterium* spp. are the third most common HACEK microorganism implicated in HACEK endocarditis [1]. Few reports of extra-cardiac *Cardiobacterium* spp. infection have been described [2, 3], and there are no known reports of the *C. valvarum* species being implicated in osteomyelitis.

Herein, we report a case of *C. valvarum* aortic endograft infection, which was further complicated by vertebral discitis, epidural phlegmon, septic cerebral emboli, and the first known case of *C. valvarum* osteomyelitis. A review of the English-language literature follows the case presentation.

## Case presentation

In June 2020, a 57-year-old man was directly admitted to the hospital for radiologic evidence of vertebral osteomyelitis, discitis, and epidural phlegmon. He had a history of hypertension, hyperlipidemia, prediabetes, chronic kidney disease, and an acute Stanford Type A and Type B aortic dissection. His Type A aortic dissection (defined as involvement of the ascending aorta) had led to an emergent resection and replacement of the ascending aorta and proximal arch with a Dacron endograft nine years prior to his current presentation. Subsequent progression of an associated aortic arch aneurysm required surgical revision with a Dacron endograft exchange five years after initial surgical repair. His Type B dissection (defined as being limited to the aorta distal to the left subclavian artery without involvement of the ascending aorta) extended from the proximal aortic arch to the aortic bifurcation and remained untreated, though partially thrombosed. He had no other recent surgical instrumentation and his last dental procedure was ~ 24 months prior to his current presentation. Medications included amlodipine, aspirin, atorvastatin, carvedilol, ezetimibe, and lisinopril. He used alcohol socially and had never used tobacco or illicit drugs.

**Fig. 1:**
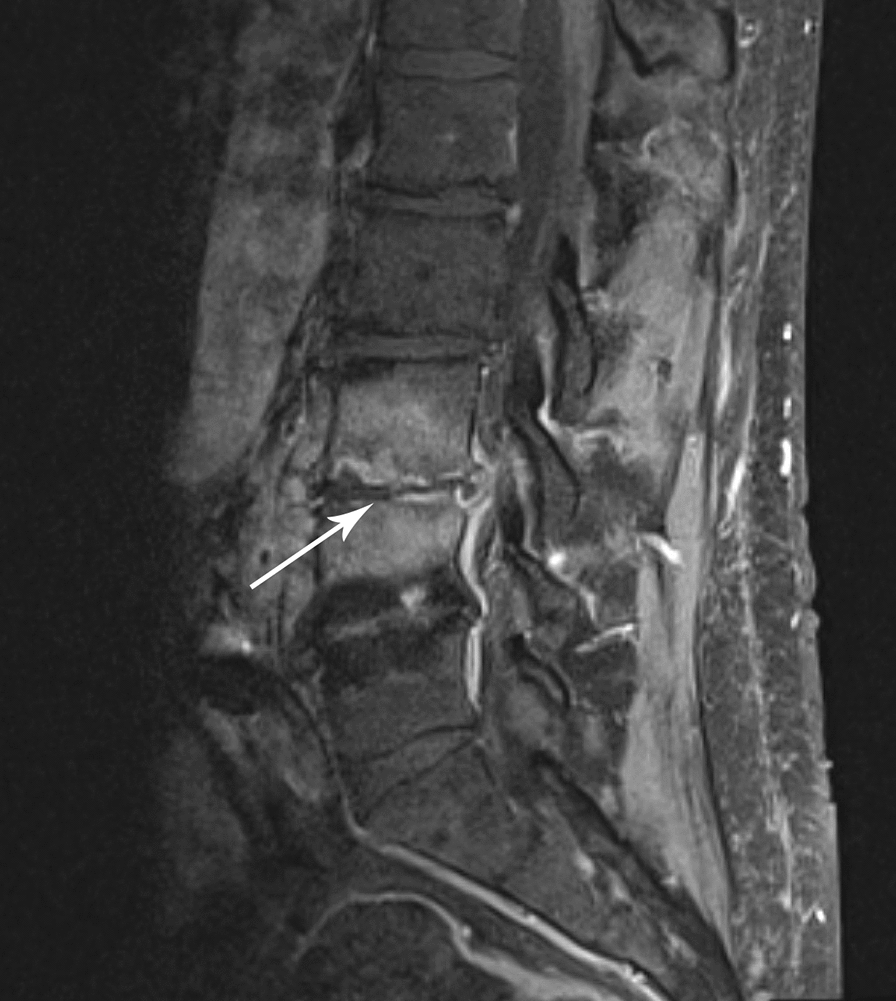
Magnetic resonance imaging (MRI) of the lumbar spine demonstrating discitis and vertebral osteomyelitis. T1 post-contrast sagittal MRI demonstrating enhancement at the L3–4 intervertebral disc space with erosions in the adjacent endplates (arrow)

The patient had been well until ~ 6 weeks prior when isolated lower back pain developed. At that time, an outpatient non-contrast-enhanced magnetic resonance imaging (MRI) of the lumbar spine demonstrated significant edema within the vertebral bodies adjacent to the L4–L5 intervertebral disc space. Given his absence of systemic symptoms, short-term follow-up was advised. Two weeks later, a repeat contrast-enhanced MRI of the lumbar spine demonstrated progressive T2 hyperintensity at the L4–L5 intervertebral disc space and adjacent endplates with near complete involvement of the L3 and L4 vertebral bodies, highly suggestive of vertebral osteomyelitis and discitis (Fig. 1a). In addition, there was a 6 mm thick anterior epidural thickening and enhancement suggestive of epidural phlegmon that extended to L3 superiorly, S1 inferiorly, and the posterior wall of the known abdominal aortic aneurysm anteriorly (Fig. 1b). He was admitted for further workup and management.

On admission, a temperature of 37.2 °C, heart rate of 74 beats per min, blood pressure of 129/67 mm Hg, and body-mass index of 32.7 were observed. On examination, there were multiple dental amalgam fillings and a 3/6 systolic murmur best appreciated in the aortic region, which was stable from prior. The remainder of the examination, including a focused neurologic, musculoskeletal, and dermatologic assessment, was normal. Basic laboratory tests were remarkable for anemia of inflammation, a C-reactive protein of 1.6 mg/dL (reference range, 0.0–0.5 mg/dL), an erythrocyte sedimentation rate of 53 mm/h (reference range, 3–10 mm/h), and the absence of leukocytosis. Aerobic and anaerobic peripheral blood cultures were obtained, and empiric vancomycin 1 g intravenously every 12 h and cefepime 2 g intravenously every 12 h were administered due to the proximity of the epidural phlegmon to the posterior wall of the known abdominal aortic aneurysm.

**Fig. 2:**
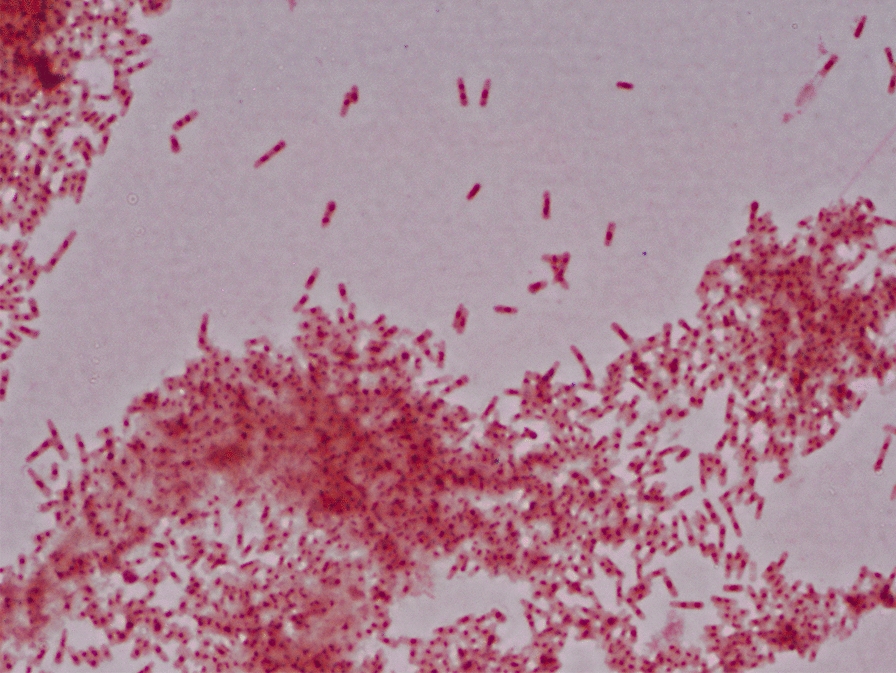
Gram staining. Microscopic morphology in gram staining of blood culture after 96 h of aerobic incubation at 37 °C demonstrating bipolar-staining gram-negative bacilli. 16S ribosomal RNA (rRNA) sequencing subsequently identified the organism as *Cardiobacterium valvarum*

A series of diagnostic tests were subsequently performed. Computed tomography (CT) angiography of the thoracic aorta showed a soft tissue density located on the ascending aortic endograft, favored to be infectious in etiology. Transesophageal echocardiogram showed previously noted strand-like mobile echodensities on the right coronary cusp of the aortic valve, favored to represent Lambl’s excrescences (defined as thin, fibrinous structures at the lines of valve closure and postulated to be related to shear forces to the endothelium) [4]. There were no other valvular abnormalities or signs of valvular insufficiency. Contrast-enhanced CT of the face showed no evidence of dental disease. Fluoroscopy-guided bone biopsy of the L4 vertebral body obtained on day 3 of admission showed changes suggestive of resolving osteomyelitis. On day 4 of admission, 2 of 2 bottles on both sets of initial peripheral blood cultures signaled positive at 96 h of incubation. Macroscopically, colonies were described as irregular, small, round, and opaque in color. Nevertheless, due to poor growth secondary to the fastidious nature of the organism, susceptibilities were unable to be performed. Bipolar-staining pleomorphic Gram-negative rod-shaped bacilli were seen on microscopy (Fig. 2). 16S ribosomal RNA sequencing analysis subsequently identified *C. valvarum* (see Appendix 1 for further information regarding methods)*.* Matrix-assisted laser desorption/ionization time-of-flight (MALDI-TOF) mass spectrometry for further identification was not performed. Aerobic and anaerobic vertebral biopsy cultures remained negative and 16S ribosomal RNA sequencing was unfortunately not performed on these specimens. Though not definitively documented in the bone, *C. valvarum* identified in the blood was presumed to be the cause of the focal lesion as well.

Antimicrobial therapy was changed to ceftriaxone 2 g intravenously daily and prescribed for a 6-week course. Given radiologic evidence of a possible aortic endograft nidus, a vascular surgery evaluation was obtained. Surgical intervention was ultimately deferred due to the high likelihood of extensive endograft involvement and the technical difficulty posed by the patient’s complex surgical history. The patient was discharged home on day 9 of admission with multidisciplinary follow-up and outpatient antimicrobial therapy.

**Fig. 3:**
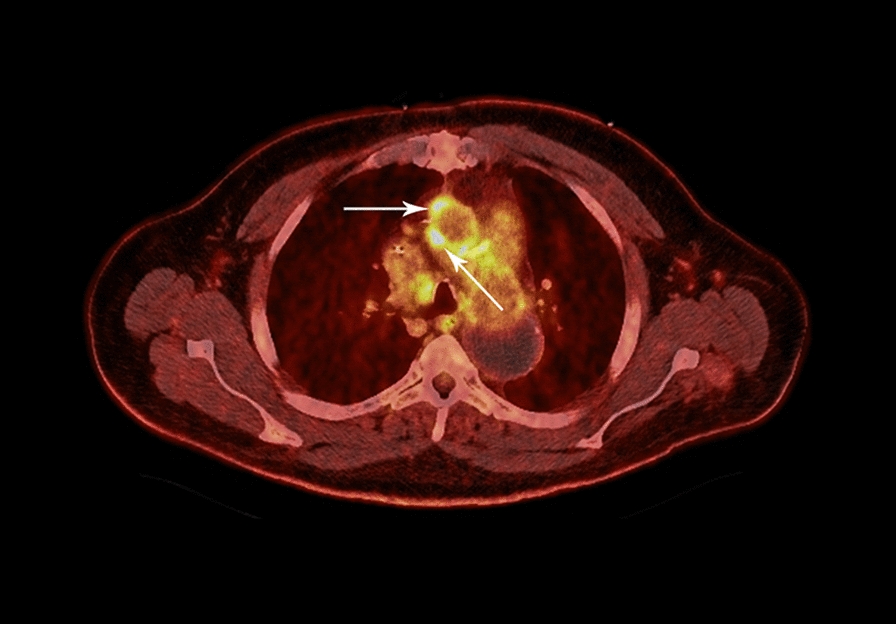
Positron emission tomography–computed tomography (PET/CT) of the chest. Hypermetabolic soft tissue (arrows) along the right lateral and anterior aspect of the ascending aortic endograft with a maximum standardized uptake value of 9.4, suspicious for aortic endograft infection

Twenty days later, the patient was readmitted for acute headache and blurry vision in the left eye. MRI of the brain at that time showed a moderate-sized acute and subacute right posterior cerebral artery (PCA) territory infarct of the right occipital and posterior temporal lobes with a small focus of associated hemorrhagic transformation. Magnetic resonance angiography of the brain subsequently showed poor flow-related signal of the right PCA P3/P4 segment with a paucity of vessels in the right occipital lobe suspicious for occlusion. Monitoring of the bilateral PCAs with a 2-MHz transcranial doppler ultrasound showed nine microembolic signals within the right PCA, suspicious for septic emboli. No neurosurgical intervention was pursued, initial antithrombotic therapy was held, and repeat peripheral blood cultures were negative. Positron emission tomography–computed tomography (PET/CT) was then obtained to better characterize the aortic endograft, which showed hypermetabolic soft tissue along the right lateral and anterior aspect of the ascending aortic graft, once again suspicious for endograft infection (Fig. 3). Surgical intervention was again deferred, and the patient was discharged home on hospital day 7. He was continued on ceftriaxone 2 g intravenously daily with the addition of clopidogrel 75 mg daily and levetiracetam 1 g twice daily. He remains well in multidisciplinary outpatient follow-up with marked improvement in his back pain and no further complication. He will remain on lifelong suppressive antimicrobial therapy following his initial treatment course.

## Discussion

The *Cardiobacterium* genus was first described in 1964 when its major and prior sole species, *C. hominis*, was recognized as a distinct microorganism by Slotnick and Dougherty [5]. *C. hominis* itself is a low-virulence, commensal and facultative Gram-negative bacillus that typically manifests in patients with subacute endocarditis. Historically, its microbiologic detection has been challenging due to its fastidious nature and slow-growth on traditional media.

In 2004, a novel *Cardiobacterium* species designated *C. valvarum* was isolated from the blood of a 37-year-old man with subacute endocarditis of a congenital bicuspid aortic valve and sudden rupture of a cerebral aneurysm [6]. In comparison to the major *C. hominis* species, *C. valvarum* grows more slowly, is non-hemolytic on sheep blood agar, and does not utilize sucrose, maltose, or mannitol on traditional media [6]. Since its initial recognition, there have been 15 total cases of *C. valvarum* infection reported in the English-language literature, all of which have utilized 16S ribosomal RNA sequencing as a means of detection [6–23]. Table 1 provides an overview of these cases. In the overwhelming majority, clinical presentations are insidious and without apparent signs of a systemic inflammatory response, such as pyrexia or chills [6, 7, 12–16]. Factors that appear to predispose patients to infection with *C. valvarum* include recent dental instrumentation [6, 10, 15, 16], poor dentition [9, 15, 18], and congenital cardiac disease [6, 8–10, 12, 13, 15–17, 19]. Importantly, the presence of a congenital bicuspid aortic valve appears to be strongly associated with *C. valvarum* pathogenicity [6, 8, 9, 13, 16, 19]. Common imaging findings in infected hosts typically include large (> 1 cm) valvular vegetations on echocardiography [6–9, 12, 16, 18, 20], and, in settings where acute neurologic dysfunction is evident, septic cerebral emboli resulting in cerebrovascular accident [6–8, 12–14, 18].

**Table 1:** Existing case reports documenting infection by *Cardiobacterium valvarum*

	Han et al. (2004)	Hoover et al. (2005)	Bothelo et al. (2006)	Gonzalez et al. (2007)	Geissdorfer et al. (2007)	Vaněrková et al. (2010)	Hoffman et al. (2010)	Chen et al. (2011)	Abraham et al. (2012)	Choudhury et al. (2013)	Pusch et al. (2015)	Bonavent et al. (2016)	Ni et al. (2018)	Irabien et al. (2019)
Patient age	37	46	51	29	71	63	28	64	65	53	45	72	41	18
Symptoms at presentation	Insidious, cardiopulmonary	Insidious	Insidious	Insidious	Insidious	Insidious	Insidious, cardiopulmonary	Insidious	Insidious, cardiopulmonary	Insidious	Cardiopulmonary	Neurologic	Cardiopulmonary	Insidious
Risk factors	Dental	Dental, cardiac	Dental, cardiac	None	Cardiac	None	Dental, cardiac	Dental, cardiac	None	Dental, cardiac	Cardiac	Cardiac	Dental	Cardiac
Cardiac imaging findings	Valvular disease, vegetations	Valvular disease, reduced LVEF	Vegetations, Abscess	Valvular disease, aortic dehiscence	Valvular disease, vegetations, abscess	Valvular disease, vegetations	Valvular disease, vegetation	Valvular disease	Valvular disease, vegetations	Valvular disease	Vegetations	Valvular disease, vegetations	Valvular disease, vegetations	Valvular disease
Neurologic symptoms	Facial Droop	None	None	Slurred speech, numbness	Weakness	None	None	None	Dysphasia, hemiplegia, facial weakness, Babinski’s sign	Headache	None	Vision loss, confusion	None	None
CNS imaging findings	SAH	None	None	Acute infarcts	Acute infarcts	None	None	None	vasculitis, acute infarct	Acute infarcts	None	Acute infarct	SAH, acute infarct	None
Surgical intervention	AVR	AVR	AVR	AVR	AVR	AVR, TVA	Replacement Prosthetic Valve-conduit	MVR	None	AVR	None	AVR	MVR, TVA	Repair MV, TV, ASD, RVOT Conduit
Antimicrobial treatment	Piperacillin-tazobactam	Ceftriaxone	Amoxicillin, gentamicin	Ceftriaxone, vancomycin	Ceftriaxone, rifampin, amikacin	Ceftriaxone, gentamicin, cefuroxime	Ceftriaxone	Cefuroxime, gentamicin	Gentamicin, piperacillin-tazobactam	Ceftriaxone, levofloxacin	Ceftriaxone	Penicillin, gentamicin, moxifloxacin	Ceftizoxime	Ceftriaxone
Clinical outcome	Alive	Alive	Unknown	Alive	Deceased	Alive	Alive	Alive	Deceased	Alive	Alive	Alive	Alive	Alive

*CNS* indicates central nervous system, *LVEF* left ventricular ejection fraction, *SAH* subarachnoid hemorrhage, *AVR* aortic valve replacement, *MVR* mitral valve replacement, *TVA* tricuspid valve annuloplasty, *MV* mitral valve, *TV* tricuspid valve, *ASD* atrial septal defect, *RVOT* right ventricular outflow tract

In the present case, *C. valvarum* bacteremia was suspected to have caused an endograft infection, later spreading to the bone. The presumed source of *C. valvarum* was the oral cavity given that the patient had undergone at least one dental procedure in the interim between his aortic arch surgical revision and the onset of lower back pain. While it is unclear if the patient was ever prescribed prophylactic antibiotic therapy prior to any known dental procedure, one possibility is that transient bacteremia resulting from one of these procedures seeded the aortic Dacron endograft. Transient bacteremia may also have resulted from manipulation associated with toothbrushing. The endograft may have then served as a nidus for recurrent episodes of transient bacteremia, leading to lumbar spine infection with subsequent development of osteomyelitis and contiguous spread into the epidural and intervertebral disc spaces. It should be noted that a single report of *C. valvarum* aortic endograft infection confirmed by 16S ribosomal RNA sequence analysis has been previously described in a 53-year-old man with total aortic arch replacement and severe aortic regurgitation in Japan [14]. Likewise, a *Cardiobacterium* spp (*C. hominis*) causing vertebral osteomyelitis, discitis, and epidural abscess has also been described [21]. To our knowledge, the present case describes the first known case of *C. valvarum* both affecting an aortic endograft and resulting in osteomyelitis. It should be noted that, in our case, vertebral bone biopsy cultures did not grow any organisms, although the *C. valvarum* identified in the blood was presumed to be the cause of the osteomyelitis as well.

In any patient with confirmed *Cardiobacterium* spp. infection, careful evaluation for the presence of infective endocarditis is mandatory given the high likelihood of cardiac involvement. In contrast to the majority of cases shown in Table 1 that met modified Duke criteria for “Definite” infective endocarditis, the present case was designated as “Possible” infective endocarditis by only meeting one major clinical criterion (blood cultures with a typical infective endocarditis microorganism) and two minor clinical criteria (predisposing cardiac condition and vascular phenomena) [22]. Notably, there was no echocardiographic evidence of endocardial or valvular involvement on both transthoracic and transesophageal studies. While mobile strand-like echodensities were noted on aortic valve imaging, these were favored to represent Lambl’s excrescences [4] and had been noted on previous echocardiographic examinations that predated his aortic dissection. Therefore, though rare, it appears that *C. valvarum* has the potential for pathogenicity in the absence of cardiac involvement.

Antimicrobial therapy is the cornerstone of management for all *C. valvarum* infections, and surgical consultation is warranted in cases with an obvious radiologic and/or echocardiographic nidus. The standard antimicrobial therapy for HACEK group microorganisms in existing guidelines is ceftriaxone 2 g intravenously daily for 4 weeks [23], and most reports have successfully utilized this regimen [10, 13, 15–17, 19]. The empiric choice of antimicrobial therapy has varied in published cases (Table 1), which is likely explained by local patterns of resistance, country-specific practices, and the presence or absence of embolic disease. Given the rarity of extra-cardiac manifestations, no guidelines dictate treatment of *C. valvarum* infection in this setting. Overall, *C. valvarum* appears to be susceptible to most antibiotics, with low minimum inhibitory concentrations to penicillins, cephalosporins, fluoroquinolones, and aminoglycosides [6–8, 10, 12].

## Conclusions

We report a case of *C. valvarum*-associated aortic endograft infection, which was further complicated by vertebral discitis, epidural phlegmon, septic cerebral emboli, and the first known case of *C. valvarum* osteomyelitis. *C. valvarum* is a rare and emerging HACEK group microorganism that warrants consideration in high-risk patients with evidence of subacute infection and disseminated disease. Most cases have successfully been treated with a third-generation cephalosporin. Source control may not be feasible in cases with complex anatomy and disseminated spread.

## Data Availability

All data generated or analyzed during this study are included in this published article.

## Appendix 1

One colony of organism was suspended in 50 μl of water and boiled at 100 °C for 10 min. The cell lysate was then centrifuged at 12,000 × *g* for 5 min to precipitate cellular debris, and the supernatant was transferred to a new sterile tube. Polymerase chain reaction amplification and sequencing of the 860 bp fragment of 16S rRNA gene was performed with the primers 5′-GAGTTTGATYMTGGCTCAGRRYGAACGCT-3′ and 5′-GACTACCAGGGTATCTAATCC-3′, corresponding to *E. coli* 16S rRNA positions 9–30 and 804–783, respectively. Identification of organism was determined by matching with sequences from GenBank with the top score.

## Abbreviations

CT: Computed tomography
HACEK: *Haemophilus* Spp., *Aggregatibacter* spp., *Cardiobacterium* spp., *Eikenella corrodens*, and *Kingella* spp.
MRI: Magnetic resonance imaging
PCA: Posterior cerebral artery
